# Stress Hyperglycemia Ratio as a Predictor of In-Hospital Stent Thrombosis in STEMI Patients Undergoing Primary PCI: A Retrospective Cohort Study

**DOI:** 10.3390/medicina61071158

**Published:** 2025-06-26

**Authors:** Evliya Akdeniz, Cennet Yıldız, Mehmet Karaca, Mehmet Pişirici, Hasan Ali Sinoplu, Onur Akpınar, Atakan Arpac, Didem Mirgün Manioğlu, Dilay Karabulut, Fatma Nihan Turhan Çağlar

**Affiliations:** 1 Department of Cardiology, Bakırköy Dr. Sadi Konuk Training and Research Hospital, 34147 Istanbul, Turkey; cennet_yildiz@live.com (C.Y.); pisiricimehmet@gmail.com (M.P.); hasanalisinoplu@gmail.com (H.A.S.); dr.akpinaronur@gmail.com (O.A.); atakan.arpac@gmail.coom (A.A.); didemmrgn.1@gmail.com (D.M.M.); dilay_karakozak@hotmail.com (D.K.); nhnturhan@gmail.com (F.N.T.Ç.); 2 Department of Cardiology, Ataşehir Memorial Hospital, 34750 Istanbul, Turkey; mehmetkaraca06@gmail.com

**Keywords:** stress hyperglycemia ratio, STEMI, ST-elevation myocardial infarction, primary PCI, stent thrombosis

## Abstract

*Background and Objectives*: and Objectives: Admission hyperglycemia (AH) is common in acute ST-elevation myocardial infarction (STEMI) and linked to poor prognosis. The stress hyperglycemia ratio (SHR) reflects relative hyperglycemia and may more accurately predict outcomes. This study examined AH, SHR, and in-hospital stent thrombosis (ST) in STEMI patients undergoing primary percutaneous coronary intervention (p-PCI). *Material and Methods*: Retrospective analysis included 1034 patients. AH was defined as glucose ≥ 11.1 mmol/L at admission. SHR was calculated as admission glucose divided by estimated average glucose derived from hemoglobin A1c (HbA1c). The primary outcome was in-hospital stent thrombosis. Patients were grouped by the occurrence of in-hospital ST. Univariable, multivariable, and LASSO (Least Absolute Shrinkage and Selection Operator) logistic regression identified predictors of ST. *Results*: In-hospital ST occurred in 1.5% of patients. ST patients had higher Killip class, heart rate, white blood cell, platelet counts, creatinine, AH, and SHR. SHR was an independent predictor of ST (OR 3.15, 95% CI 1.88–5.27, *p* < 0.001), whereas AH was not (*p* = 0.182). Neutrophil count, correlated with WBC, was also a significant risk factor. ROC analysis showed SHR ≥ 1.26 as an optimal cutoff predicting ST. *Conclusions*: SHR is a strong independent predictor of in-hospital ST after STEMI, superior to AH. Monitoring and managing stress-induced hyperglycemia play a crucial role in the setting of STEMI. Further studies are needed.

## 1. Introduction

It is now well established that admission hyperglycemia (AH) in patients presenting with acute ST-elevation myocardial infarction (STEMI) is associated with unfavorable clinical outcomes. This detrimental effect of AH has been demonstrated regardless of diabetes status [1,2,3]. Although the reported incidence of AH varies among different studies, it has been observed in up to 60% of patients with acute myocardial infarction (MI), highlighting its clinical relevance and the need for early recognition and management [4,5].

The concept of relative hyperglycemia (RH), calculated using the stress hyperglycemia ratio (SHR), was first introduced by Roberts et al. as a novel measure of prognosis in patients with critical illness. Roberts et al. showed that in critically ill patients, the relative hyperglycemic response is a more clinically significant prognostic parameter than absolute hyperglycemia [6]. In the following years, clinical studies were conducted showing that the relative hyperglycemic response predicted adverse outcomes in both diabetic and non-diabetic patients with different clinical conditions [7,8,9]. Despite technological advancements and improvements in operator experience in contemporary interventional cardiology, the incidence of stent thrombosis (ST) remains as high as 2–4.5% [10,11,12]. In addition to this relatively high incidence, in-hospital mortality in patients with ST is also approximately 5%, which might be considered a significantly high rate [13]. Considering both its incidence and the severity of its clinical outcomes, ST plays a pivotal role in cardiology practice. According to the definition of the Academic Research Consortium, early ST is defined as ST occurring between 0 and 30 days after stent implantation [14]. Risk factors for early ST include several parameters such as STEMI, multi-vessel coronary artery disease, reduced left ventricle ejection fraction (LVEF), Killip classification, diabetes mellitus (DM), smoking, anemia, thrombocytosis, non-compliance with dual antiplatelet therapy, poor pre-procedural TIMI flow, thrombus burden, inflammatory status, and malignancy [15,16,17,18].

Intensive glycemic control is no longer routinely used in hospitalized patients due to the increased risk of hypoglycemia and mortality shown in the NICE-SUGAR trial. As a result, the American Diabetes Association recommends a more conservative target blood glucose range of 140–180 mg/dL. This shift in practice raises important questions about how to manage relative hyperglycemia in acute cardiovascular settings such as STEMI [19,20].

In our study, we evaluated the relationship between AH and SHR, and in-hospital ST among STEMI patients undergoing primary percutaneous coronary intervention (p-PCI).

## 2. Materials and Methods

### 2.1. Study Population

Between 2022 and 2025, patients who were hospitalized with a diagnosis of STEMI and treated with p-PCI after emergency coronary angiography were included in our study, retrospectively. The inclusion criteria were age to be older than 18 years and to be treated with p-PCI. Exclusion criteria were refusal to participate, renal failure with a glomerular filtration rate of less than 30 mL/min, failed p-PCI or balloon angioplasty only, or patients in whom intracoronary thrombus could not be demonstrated by coronary angiography due to sudden death while being monitored in the coronary intensive care unit after p-PCI.

Baseline demographic features and clinical and laboratory variables were obtained from hospital information system. Laboratory data, including glucose and HbA1c levels, were obtained from tests performed at the time of admission. LVEF was obtained by echocardiography with the Modified Simpson’s method after the p-PCI procedure while the patients were in coronary intensive care unit. The study cohort was categorized into two groups: in-hospital ST group and control group. The predictors of in-hospital ST were subsequently analyzed and determined.

Ethical approval for this study was granted by the Ethics Committee of our tertiary care institution. The research was carried out in compliance with the principles outlined in the Declaration of Helsinki.

### 2.2. Definitions

AH was defined as a blood glucose level of ≥11.1 mmol/L (≥200 mg/dL) measured at admission. RH was defined by the SHR, which was calculated by dividing the admission blood glucose by the estimated average glucose (eAG) [6]. eAG serves as a surrogate indicator of the average blood glucose level during the preceding 2–3 months and is commonly derived from HbA1c values. Nathan et al. [21] proposed an equation to estimate eAG from HbA1c levels. The equation is as follows: eAG = (1.59 × HbA1c) − 2.59.

In-hospital ST was defined in patients monitored following p-PCI is characterized by the onset of ischemic symptoms and/or clinical signs and electrocardiographic changes suggesting of MI, accompanied by angiographic evidence of a peri-stent thrombus formation [14].

### 2.3. Statistical Analysis

For continuous variables, data were reported as mean ± standard deviation, and categorical variables were described using the number and percentage. Patients with and without in-hospital ST were compared using an independent Student’s *t*-test and a Mann–Whitney U test. A receiver operating characteristic (ROC) curve was generated to assess the predictive performance of SHR and AH for in-hospital ST. Univariable logistic regression analysis was performed to identify independent predictors of in-hospital ST. Variables that were statistically significant in the univariable analysis were entered into the multivariable logistic regression analysis. Since white blood cell (WBC) and Neutrophil (NEU) counts were positively correlated, only the NEU count was entered into the multivariable analysis. LASSO (Least Absolute Shrinkage and Selection Operator) regression was performed to identify the most important predictors of in-hospital ST to prevent overfitting and improve predictive performance. All analyses were performed using SPSS statistical software version 25.0 and R statistical software version 4.5.0.

### 2.4. Endpoint

The primary endpoint of our study was in-hospital ST.

## 3. Results

This retrospective study included 1034 consecutive patients with a mean age of 59.92 ± 12.87 years; 21.1% were female. Hypertension and DM were present in 48% and 28.3% of patients, respectively, while 52.2% were active smokers. The in-hospital ST rate was 1.5% of the cohort. The baseline characteristics, along with the clinical and laboratory features of the study population and comparison between groups, are summarized in Table 1.

Upon comparison of the group with ST to the control group, non-significant differences were observed in terms of age, gender, hypertension, DM, or smoking status. Killip classification, heart rate, WBC, and platelet (PLT) count, and creatinine level were significantly higher in the ST group. In the ST group, admission glucose levels were higher (17.8 ± 9.5 mmol/L vs. 9.1 ± 5.0 mmol/L, *p* < 0.001), with a greater proportion of patients having glucose ≥ 11.1 mmol/L (56.3% vs. 20.8%, *p* = 0.002). The SHR levels were significantly higher in the ST group (2.17 ± 0.98 vs. 1.09 ± 0.30, *p* < 0.001). Both AH and SHR were higher in the ST group compared to the control group. The comparative data of the two groups are presented in Table 1.

Univariate analysis identified several significant factors associated with ST. KILLIP class 3–4 was strongly linked to ST (OR: 7.332, 95% CI: 2.596–20.713, *p* < 0.001). Additionally, higher heart rate, WBC, PLT, admission glucose level, and serum creatinine were associated with increased ST risk. Glucose levels ≥ 11.1 mmol/L (OR: 4.888, 95% CI: 1.800–13.277, *p* = 0.002) and the SHR (OR: 23.232, 95% CI: 8.900–60.644, *p* < 0.001) seemed to be predictors of in-hospital ST (Table 2). Multivariate logistic regression analysis was conducted to identify independent predictors of in-hospital ST. While AH was not found to be a statistically significant predictor (OR: 2.210, 95% CI: 0.690–7.076, *p* = 0.182), SHR emerged as an independent and significant predictor of in-hospital ST (OR: 22.301, 95% CI: 5.471–90.908, *p* < 0.001). Furthermore, it was revealed that NEU, which correlates with WBC, is also a significant risk factor (Table 3). Accordingly, we conducted LASSO regression to identify the most important predictors of in-hospital ST, aiming to reduce overfitting and improve predictive performance. SHR was verified as a strong and significant predictor, with an Odds Ratio of 3.15 (95% CI: 1.88–5.27, *p* < 0.001) (Table 4).

**Figure 1:**
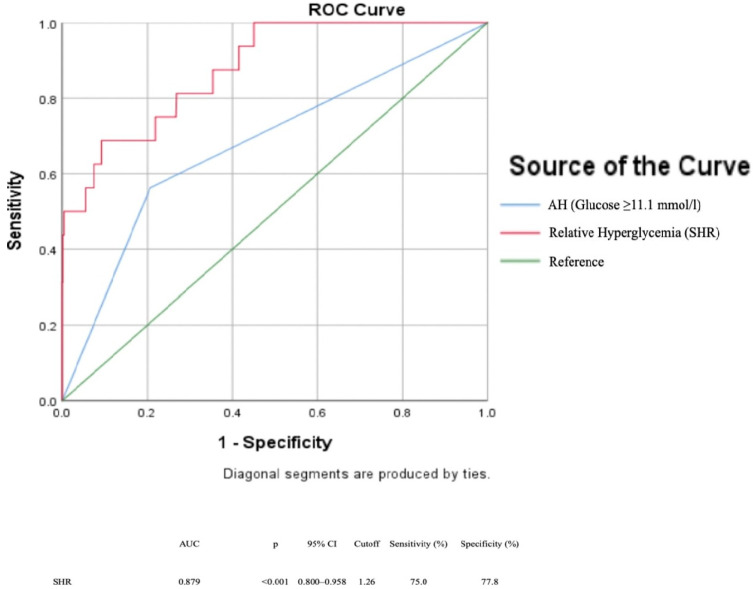
Receiver operator characteristic (ROC) curve for SHR predicting in-hospital ST. AH: admission hyperglycemia, AUC: Area Under Curve, SHR: stress hyperglycemia ratio. AUC = 0.879 (95% CI: 0.800–0.958), cutoff ≥ 1.26 yielded 75% sensitivity and 77.8% specificity.

ROC curve analysis for the SHR demonstrated a strong predictive ability for in-hospital ST, with an area under the curve (AUC) of 0.879 (95% CI: 0.800–0.958, *p* < 0.001). The optimal cut-off value of SHR for in-hospital ST was determined to be ≥ 1.26, yielding a sensitivity of 75% and a specificity of 77.8% (Figure 1).

## 4. Discussion

The key results of our retrospective cohort study are summarized as follows: First, the SHR is a robust and independent predictor of in-hospital ST in patients with STEMI undergoing p-PCI. Second, although the AH rate appeared markedly higher in the ST group compared to the control group (56.3% vs. 20.8%, respectively), it was not found to be a statistically significant independent predictor of in-hospital ST. Third, an SHR value ≥ 1.26 independently and strongly predicts the risk of in-hospital ST. The latter data are in line with the findings of the study by Chu et al., where an SHR value of ≥1.19 was identified as a predictor of intracoronary thrombus burden in STEMI patients [22]. Although HbA1c is an important marker that provides information about glucose metabolism over the past three months, it can be affected by both acute and chronic conditions such as acute blood loss, blood transfusion, severe renal failure, and chronic liver failure [23,24]. This should also be taken into consideration in patients with acute myocardial infarction.

In the overall cohort population, the proportion of diabetic patients was 28.3%, and this rate was similar between the ST group and the control group. Although diabetic patients tend to have a greater predisposition to hyperglycemia compared to non-diabetics, multiple studies have reliably identified a strong association between the SHR and negative clinical outcomes among patients presenting with acute coronary syndrome, across both diabetic and non-diabetic cohorts [25,26,27]. These scientific findings constitute the main rationale for not stratifying our entire study cohort based on the presence or absence of diabetes. Although KILLIP class did not reach statistical significance as an independent predictor of in-hospital ST in the multivariate analysis, the proportion of patients presenting with KILLIP class 3 and 4 was higher in the ST group compared to the control group—3.6% vs. 12.5% and 3.9% vs. 25%, respectively. Following acute MI, diastolic dysfunction characterized by elevated left ventricular filling pressures can lead to dysfunction of the coronary microcirculation. As a result, the flow velocity in the epicardial coronary arteries may be relatively reduced, which can explain the higher incidence of ST observed in KILLIP class 3–4 patients [28].

Although the underlying pathophysiological mechanisms by which hyperglycemia serves as a poor prognostic marker in STEMI patients are not clearly understood, several potential mechanisms can be suggested. Firstly, Chu et al. [22] demonstrated that the SHR could predict a large thrombus burden in patients with STEMI. Considering that acute hyperglycemia has been scientifically proven to augment thrombin formation and promote platelet activation, it appears plausible that stress hyperglycemia—rather than chronic hyperglycemia—may contribute to a higher thrombus burden. In this context, as demonstrated in our study, the hypothesis that SHR facilitates in-hospital ST is consistent with existing scientific evidence [29,30]. Another parameter that may explain the association between hyperglycemia and poor prognosis is infarct size. Lønborg et al., using cardiac magnetic resonance imaging, demonstrated that acute hyperglycemia is associated with a larger infarct size and a greater area of myocardium at risk [2]. The third potential mechanism contributing to the pathogenesis may be the enhancement of oxidative stress and microvascular dysfunction induced by acute hyperglycemia [31]. It is also well known that stress hyperglycemia induces a proinflammatory state in patients with AMI [32].

When all of the aforementioned mechanisms are considered together, the prothrombotic and proinflammatory effects of stress hyperglycemia—along with its propensity to enhance oxidative stress and predispose to microvascular dysfunction, which may facilitate the development of the no-reflow phenomenon (a condition that has been shown to be associated with stress hyperglycemia)—may lead to an expansion of the infarcted myocardial area [33]. This combination of pathophysiological mechanisms offers a reasonable explanation for the link between acute hyperglycemia and poor clinical outcomes in patients with MI. SHR, as an independent predictor of in-hospital ST, may reflect the adverse effects of acute hyperglycemia—namely, the induction of a prothrombotic and proinflammatory state along with microvascular dysfunction—which together may lead to impaired coronary blood flow and, subsequently, increase the risk of thrombotic complications.

In recent years, metabolic and immunity-based biomarkers have gained significant importance in cardiology practice [34]. Based on the concept that stress-induced hyperglycemia is associated with poor clinical outcomes due to its prothrombotic and proinflammatory effects, provide a rationale for future clinical studies investigating whether the close monitoring and management of stress hyperglycemia may lead to improved clinical outcomes.

### Limitations

Our study has a number of limitations that must be taken into account. First, its retrospective design might facilitate potential biases, including selection and information bias. Second, the relatively small number of patients who developed in-hospital ST may have limited the statistical power to detect other potential predictors or interactions. Third, our study focused exclusively on in-hospital ST; therefore, due to the absence of early and long-term post-discharge follow-up data, the potential impact of SHR on the risk of ST after hospital discharge could not be evaluated. Additionally, in-hospital glycemic control strategies were not evaluated, and thus, the potential impact of therapeutic interventions could not be taken into account. Although the prevalence of ST is reported to be between 2 and 4.5% worldwide, it was found to be 1.5% in our study, which is slightly lower but still comparable to those reported in the literature. However, since the number of patients who developed in-hospital ST in our study was only 16, subgroup analysis dividing ST patients into diabetic and non-diabetic groups could not be performed. Another important limitation to address is that our study included only patients who underwent successful p-PCI. However, potential confounding factors such as stent type and size, high thrombus burden, dissection, or multivessel disease should be considered, as they may contribute to the development of in-hospital ST.

## 5. Conclusions

In conclusion, we identified the SHR as a strong and objective risk factor of in-hospital ST in patients with STEMI undergoing p-PCI. To the best of our knowledge, few studies have specifically evaluated the relationship between SHR and in-hospital ST. An SHR threshold of ≥ 1.26 was significantly associated with increased incidence of in-hospital ST, underscoring the clinical applicability of SHR within this group of patients. While AH was more prevalent among patients who developed ST, it did not independently predict this outcome. Therefore, our findings emphasize the prognostic relevance of SHR and suggest that closer glycemic monitoring and control during the acute phase of STEMI may be beneficial in reducing in-hospital ST. Furthermore, although an SHR cutoff of ≥ 1.26 was identified as predictive of in-hospital ST, this threshold requires validation in larger, prospective, and multi-center cohorts to ensure its generalizability.

## Figures and Tables

**Table 1:** Baseline characteristics of the study population and comparison of clinical characteristics between control group and in-hospital ST group.

Variable	Overall (n = 1018) (Mean ± SD or n (%))	Control Group (n = 1018)	In-Hospital ST Group (n = 16)	*p*-Value
Age (years)	59.92 ± 12.87	59.93 ± 12.83	55.94 ± 14.35	0.130
Female gender	218 (21.1%)	215 (21.1%)	3 (18.8%)	0.818
HTN	496 (48%)	490 (48.1%)	6 (37.5%)	0.395
DM	293 (28.3%)	290 (28.5%)	3 (18.7%)	0.666
Smoking	540 (52.2%)	530 (52.1%)	10 (62.5%)	0.084
Killip Class				<0.001
Killip 1	936 (90.5%)	926 (91%)	10 (62.5%)	
Killip 2	15 (1.5%)	15 (1.5%)	0 (0%)	
Killip 3	39 (3.8%)	37 (3.6%)	2 (12.5%)	
Killip 4	44 (4.3%)	40 (3.9%)	4 (25%)	
SBP (mmHg)	146.65 ± 30.13	136.97 ± 30.03	119.87 ± 31.67	0.073
Heart Rate (bpm)	82.85 ± 20.92	82.66 ± 20.79	95.00 ± 25	0.021
LVEF (%)	45.76 ± 11.15	45.82 ± 11.16	43.57 ± 8.64	0.320
HDL (mg/dL)	35.62 ± 10.17	35.58 ± 10.18	38.25 ± 9.72	0.284
LDL (mg/dL)	113.91 ± 39.07	113.88 ± 38.78	115.57 ± 53.54	0.876
HbA1c (%)	6.69 ± 1.78	6.69 ± 1.78	6.73 ± 1.66	0.757
WBC (×10^3^/µL)	12.52 ± 4.86	12.39 ± 4.54	20.51 ± 12.51	0.004
HGB (g/dL)	13.61 ± 1.98	13.61 ± 1.97	14.31 ± 2.50	0.064
PLT (×10^3^/µL)	237.50 ± 65.99	236.55 ± 65.63	293.69 ± 66.62	0.001
hs-cTnT (ng/L)	1799.66 ± 7928.45	1765.12 ± 7903.88	4224.41 ± 9644.20	0.334
Baseline Glucose (mmol/L)	9.3 ± 5.2	9.1 ± 5.0	17.8 ± 9.5	<0.001
Creatinine (mg/dL)	1.00 ± 0.68	0.99 ± 0.67	1.37 ± 0.94	0.011
AH	221 (21.3%)	212 (20.8%)	9 (56.3%)	0.002
SHR	1.12 ± 0.36	1.09 ± 0.30	2.17 ± 0.98	<0.001

AH: admission hyperglycemia, DM: diabetes mellitus, HbA1c: hemoglobin A1c, HDL: High-Density Lipoprotein, HGB: hemoglobin, hs-cTnT: High Sensitive Cardiac Troponin T, HTN: hypertension, LDL: Low-Density Lipoprotein, LVEF: Left Ventricular Ejection Fraction, PLT: platelet, SBP: Systolic Blood Pressure, SHR: stress hyperglycemia ratio, ST: stent thrombosis, WBC: white blood cell.

**Table 2:** Univariable binary logistic regression analysis of predictors for in-hospital ST events.

Variable	OR	95% CI	*p*
Killip 3–4	7.332	2.596–20.713	<0.001
Heart Rate	1.024	1.004–1.044	0.020
WBC	1.163	1.100–1.230	<0.001
PLT	1.010	1.004–1.015	0.001
Baseline Glucose	1.008	1.005–1.011	<0.001
Creatinine	1.386	1.001–1.920	0.049
AH	4.888	1.800–13.277	0.002
SHR	23.232	8.900–60.644	<0.001

AH: admission hyperglycemia, CI: Confidence Interval, OR: Odds Ratio, PLT: platelet, SHR: stress hyperglycemia ratio, WBC: white blood cell.

**Table 3:** Multivariable binary logistic regression analysis of predictors for in-hospital ST events—Model A and Model B.

Variable	Model A			Model B		
	OR	95% CI	*p*	OR	95% CI	*p*
Killip 3–4	2.684	0.754–9.549	0.127	3.059	0.462–20.241	0.246
Heart Rate	0.999	0.977–1.021	0.927	0.978	0.948–1.010	0.176
PLT	1.006	0.999–1.013	0.114	1.009	1.000–1.018	0.063
NEU	1.124	1.046–1.208	0.002	1.011	0.902–1.133	0.852
Creatinine	1.119	0.635–1.971	0.698	0.806	0.232–2.798	0.734
AH	2.210	0.690–7.076	0.182	—	—	—
SHR	—	—	—	22.301	5.471–90.908	<0.001

AH: admission hyperglycemia, CI: Confidence Interval, NEU: Neutrophil, OR: Odds Ratio, PLT: platelet, SHR: stress hyperglycemia ratio.

**Table 4:** LASSO regression analysis results for in-hospital ST events.

Variable	OR	95% CI	*p*
Killip Class (3–4)	1.27	0.84–1.93	0.251
Heart Rate	0.64	0.34–1.22	0.175
PLT	1.77	0.98–3.22	0.060
NEU	1.03	0.62–1.71	0.917
Creatinine	0.89	0.43–1.82	0.746
SHR	3.15	1.88–5.27	<0.001

CI: Confidence Interval, NEU: Neutrophil, PLT: platelet, OR: Odds Ratio, SHR: stress hyperglycemia ratio.

## Data Availability

The original contributions presented in this study are included in the article. Further inquiries can be directed to the corresponding author.
